# Living With Long COVID: Everyday Experiences, Health Information Barriers and Patients' Quality of Life

**DOI:** 10.1111/hex.70290

**Published:** 2025-05-14

**Authors:** Esther Ortega‐Martin, Javier Alvarez‐Galvez

**Affiliations:** ^1^ Department of General Economy (Health Sociology Area) Faculty of Nursing and Physiotherapy University of Cadiz Cadiz Spain; ^2^ Computational Social Science DataLab, University Institute for Sustainable Social Development University of Cádiz Jerez de la Frontera Spain

**Keywords:** healthcare access, health information, long covid, patient experiences, quality of life

## Abstract

**Background:**

Long COVID has considerably impacted patients' daily lives, yet qualitative insights in Spain are still scarce. This study seeks to (1) explore patients' experiences and the barriers they face, (2) analyse challenges in accessing accurate information and (3) evaluate the effects on quality of life by examining its dimensions in detail.

**Methods:**

Semi‐structured interviews were conducted with 23 participants in Spain with Long COVID. Thematic analysis was performed, investigating needs, obstacles in daily life, challenges in obtaining and understanding health knowledge and its effects on the quality of life.

**Results:**

The most frequent symptoms were chronic pain and postexercise fatigue. All individuals encountered restrictions in their daily lives, which often had financial consequences. A lack of recognition coupled with legal insecurity due to the absence of a formal diagnosis further compromised their economic stability. Stigmatisation and poor social understanding led to feelings of loneliness and distress, adding to the overall impact of the disease. Health fragmentation, lack of follow‐up and absence of coordinated multidisciplinary treatment limited specialised treatment and health information. Therefore, many patients sought information and support from online communities. However, misinformation and information overload or contradictory information generated confusion, affecting decision‐making about the management of their disease, affecting disease management and quality of life.

**Conclusion:**

The impact of Long COVID transcends physical health, pointing to economic pressure, legal uncertainty and fragmentation of care. We reveal how misinformation and a lack of guidance intensify inequities in access to reliable information. These findings underscore the need for integrated models of care, policy recognition and targeted strategies to reduce socio‐economic inequalities.

**Patient or Public Contribution:**

This study expands knowledge about the experiences of people with Long COVID in Spain. Their journeys in the healthcare system and the challenges they face are key to the analysis and findings. Patient associations supported recruitment to ensure a broad range of viewpoints.

## 1 Introduction

The COVID‐19 pandemic, caused by the SARS‐CoV‐2 virus, has claimed an estimated 7 million lives worldwide [1], profoundly affecting health systems and populations [2, 3], including the Spanish healthcare system. The disease presents a wide spectrum of symptoms, ranging from asymptomatic cases to severe cases requiring hospitalisation [4, 5]. Although many individuals fully recover, some manifest long‐lasting symptoms, even after mild cases [6, 7, 8].

Long COVID (LC) emerges as the transition from the acute to the chronic illness [9, 10]. The World Health Organization (WHO) defines LC as new or continuing symptoms 3 months after initial infection, lasting at least 2 months without explanation [11]. LC affects multiple organ systems [7, 10, 12, 13]. LC is characterised by a diverse range of symptoms (e.g., weakness, general malaise, fatigue and impaired concentration) [4, 14, 15], which often reduce the quality of life (QoL) [14, 15]. Research estimates that between 6% and 15% of individuals who had COVID‐19 continue to experience prolonged symptoms. [9, 15, 16, 17].

The pandemic brought with it a wave of misinformation that has been extensively studied [18, 19, 20, 21]. Similarly, information‐seeking behaviour has been studied in the context of chronic diseases [22, 23]. However, information seeking in the context of LC has been little, leaving a gap in understanding how they access and process information about their disease. So far, studies have explored the organisation of online communities dedicated to LC [24] but have not analysed in‐depth the information sources used or their reliability. Despite growing LC research, gaps in knowledge remain in understanding patients' health systems navigation, symptom management and access to reliable health information [25, 26, 27, 28].

Evidence indicates that many patients with LC have a significant reduction in QoL [29, 30]. Studies conducted in Spain found an impact of LC on daily life [29, 30, 31, 32, 33, 34, 35], with a significant decrease in QoL [31, 32], decreasing functionality, fatigue and lower levels of physical activity and increased utilisation of healthcare services [32]. The findings also demonstrate that LC patients also experience lowered levels of self‐perceived well‐being due to LC symptoms and several limitations in their daily lives [32, 33, 36]. However, the high variability in the combination of different conditions and personal circumstances hinders the clinical management and recovery of LC patients [37, 38]. Therefore, a thorough understanding of the life experiences and clinical pathways of these patients with complex disease patterns is critical to the provision of effective clinical care [12] and the appropriate management of associated costs.

Although some qualitative studies have been conducted in Spain [33, 35, 39, 40, 41], they focus on regional populations or specific aspects of LC, such as its emotional impact or rehabilitation, limiting their applicability at a national level. Furthermore, studies in regions such as the Basque Country [35] have not comprehensively addressed the full spectrum of QoL, particularly the intersection between dimensions such as neuropsychological symptoms and employment. In addition, little attention has been given to how these patients find health information barriers, despite the literature indicating that chronic patients are more exposed to online content due to the lack of adequate solutions provided by conventional healthcare systems. This study contributes to filling these gaps by examining QoL at the national level, providing a broader understanding of how LC impacts patients' daily lives and healthcare experiences, while also identifying specific challenges in Spain.

The impact of LC on patients' QoL has led to an urgent need for comprehensive studies from a holistic perspective to guide evidence‐based policy development and health system adaptation [40]. In this context, exploring the intersection between QoL, access to healthcare and health information barriers is vital for prioritising patient‐centred interventions and integrated healthcare strategies. This study adopts an interpretive approach to explore lived experiences and barriers within a broader social context composed of factors such as gender, socio‐economic status and institutional legitimacy of suffering.

Despite growing research on LC, relevant knowledge gaps remain, particularly in patients' experiences with healthcare navigation, symptom management and access to reliable health information. To add qualitative evidence to current knowledge, this study aims to (1) characterise the everyday experiences of LC patients and identify the social and health barriers they encounter, (2) examine the difficulties in obtaining accurate information, and its consequences for making decisions and managing the disease and (3) evaluate the impact on their QoL by thoroughly examining the various dimensions of this concept.

## 2 Materials and Methods

### 2.1 Study Design

This qualitative study used semi‐structured interviews with patients with a self‐reported diagnosis of LC living in Spain. Interviews were conducted online (via video calls) or in person, between June and November 2023.

Semi‐structured interviews captured the LC patients' life experiences until data saturation. Then, transcriptions were analysed iteratively through thematic analysis, with categories discussed and refined by the research team until consensus was reached.

A health questionnaire (Supporting Information S1) collected relevant socio‐demographic (e.g., age, sex, and educational level) and health‐related questions (e.g., healthcare consultations, disabilities). The interviews were semi‐structured, using the guide (Supporting Information S2) based on Helfferich [42]. The interviews included the themes of QoL, experiences and needs, health service use and information.

This study received approval from the Ethics Committee of Cádiz (Code: 0659‐N‐23). Participants received and independently read a document with information about the study and its objective; after reading it, they were able to ask questions about the study. Participants gave their written consent, and then the interview occurred. Anonymity and confidentiality were strictly maintained throughout the research process.

### 2.2 Recruitment of Participants

Collaboration was established with national and regional patient associations, including the Long COVID ACTS Collective (National), Long COVID ACTS Andalusian Association, Long COVID ACTS Asturias Collective, AMACOP—Madrid Association of Long COVID and Long COVID Euskal Herria. Additionally, recruitment was also facilitated through the researchers' contacts. This approach reached a broad sample of individuals affected by LC in Spain. Purposive sampling was employed, ensuring diversity in variables such as region of residence, age, gender and socio‐economic status to achieve a broad representation of experiences. Participants were recruited through phone calls, emails and social media, with support from patient associations. Informed consent was obtained from all participants.

The selection criteria included an LC diagnosis (meeting the WHO definition), age over 18, Spanish residency and fluency in Spanish. The exclusion criteria included individuals who did not meet the inclusion requirements, those unable to consent and those incapable of responding to the interview questions.

### 2.3 Qualitative Analysis

Interviews were recorded, transcribed verbatim and anonymised. Transcripts were analysed using ATLAS.ti 24.1.1 [43] software to facilitate data organisation and management. The interviews continued until theoretical saturation was reached. The qualitative thematic analysis was based on a thematic content analysis approach using Braun and Clarke's thematic analysis method [44]. Data analysis was guided by the research questions and the aim through a constructionist perspective. Initially, two trained researchers independently coded a subset of transcripts to identify emergent themes. The identified codes were then compared, discussed and refined in an iterative process to ensure coherence and consistency across the data, allowing for the emergence of rich, contextually grounded themes.

Thematic analysis was used to create the QoL dimensions inductively, ensuring that they were not preset categories but rather a product of participant narratives. This method made it possible to identify the most pertinent factors influencing patients' lived experiences using data.

## 3 Results

### 3.1 Characterisation of the LC Patients' Sample

The 23 participants (21 women and 2 men), aged between 19 and 63 years (mean = 45.30 years, SD = 8.70). Participants were distributed across mainland Spain (northern, central and southern regions), including rural and urban settings. Regarding work, 56.52% were unemployed or on sick leave, whereas 43.48% were active workers (see Table 1). Five interviewees reported permanent disabilities. In Spain, disability is assessed as a percentage by the National Social Security System. A ≥ 33% rating can grant access to specific benefits. Notably, five participants worked in the healthcare sector. On average, they reported frequent healthcare use, including visits to GPs (10.22), specialists (17.65), emergency rooms(3.65) and hospitalisations (0.61) in the past year. Most infections occurred in 2020–2021, with only three cases in 2022. The average interview time was 44.30 min (SD = 16.49).

**Table 1:** Socio‐demographic and health characteristics of patients.

ID	Age	Sex	Municipality size	Disability^a^	Work status	Co‐inhabitants	Education level	Family income (€)	Health service use			
									GP visits	Specialist visits	ER visits	Hospitalisations
E1	63	Woman	< 5000	No	Sick leave	1	Postgraduate studies	> 45,000	20	6	0	0
E2	41	Woman	100,001–500,000	No	Active	3	University degree	20,000–45,000	5	7	5	0
E3	51	Woman	100,001–500,000	Yes (33%)	Sick leave	2	Vocational training	20,000–45,000	5	20	3	0
E4	41	Woman	5001–30,000	No	Sick leave	1	Postgraduate studies	20,000–45,000	20	20	1	0
E5	42	Woman	100,001–500,000	No	Active	2	Vocational training	< 20,000	9	4	3	0
E6	43	Man	< 5000	No	Active	3	University degree	Prefer not to say	8	10	5	0
E7	46	Woman	100,001–500,000	No	Active	2	Postgraduate studies	< 20,000	15	4	0	0
E8	57	Woman	100,001–500,000	No	Sick leave	1 or 2	Vocational training	< 20,000	9	5	0	0
E9	51	Woman	> 1,000,000	No	Sick leave	1	Postgraduate studies	20,000– 45,000	12	24	1	0
E10	41	Woman	100,001–500,000	No	Unemployed	1	Vocational training	< 20,000	5	5	0	0
E11	54	Woman	100,001–500,000	No	Sick leave	0	University degree	Prefer not to say	10	21	1	1
E12	51	Woman	5001–30,000	Yes (in evaluation)	Sick leave	2	Vocational training	20,000–45,000	48	65	42	0
E13	39	Woman	100,001–500,000	In evaluation	Active	2	Postgraduate studies	> 45,000	1	31	4	0
E14	46	Woman	> 1,000,000	No	Active	5	University degree	20,000–45,000	0	12	0	0
E15	36	Woman	> 1,000,000	Yes (33%)	Active	1	High school diploma	< 20,000	2	1	0	0
E16	42	Woman	< 5000	No	Sick leave	1	Vocational training	< 20,000	24	1	4	1
E17	19	Woman	100,001–500,000	Yes (33%)	Active	4	High school diploma	> 45,000	10	40	12	12
E18	37	Woman	< 5000	No	Unemployed	2	Postgraduate studies	< 20,000	1	30	0	0
E19	47	Woman	5001–30,000	No	Sick leave	3	University degree	> 45,000	2	55	0	0
E20	49	Man	> 1,000,000	No	Active	0	Postgraduate studies	20,000–45,000	4	3	0	0
E21	45	Woman	100,001–500,000	No	Sick leave	2	Postgraduate studies	20,000– 45,000	20	15	0	0
E22	51	Woman	100,001–500,000	No	Active	4	Postgraduate studies	> 45,000	5	7	3	0
E23	50	Woman	> 1,000,000	Yes	Unemployed	0	Vocational training	< 20,000	0	20	0	0

Abbreviations: ER visits, emergency room visits; GP visits, general practitioners visits.

a In Spain, disability is assessed as a percentage by the National Social Security System.

The most reported symptoms were brain fog, joint stiffness, fever and asthenia. Health problems affecting the cardiovascular, respiratory, digestive, endocrine and dermatological systems were also mentioned during the interviews. Although most had no previous illnesses, we found some participants with multimorbidity who also reported a worsening of existing illnesses. Furthermore, patients reported the emergence of new conditions such as myalgic encephalitis, Raynaud's disease, dysautonomia and postural orthostatic tachycardia syndrome (POTS), complicating their health management.

### 3.2 Experiences

#### 3.2.1 Health Services Use

**Figure 1:**
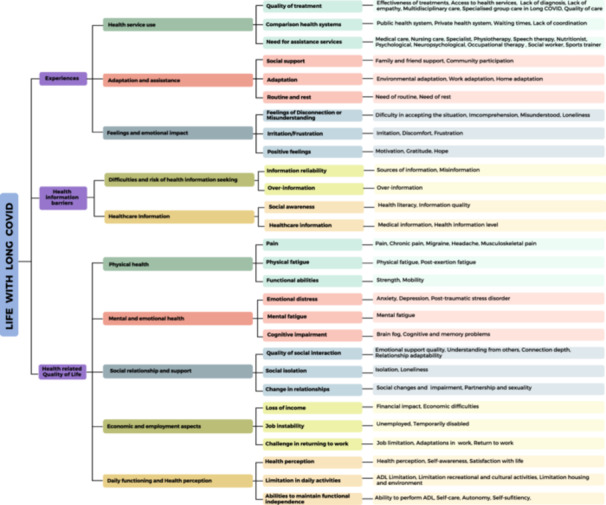
Outline of experience, quality of life and unmet needs codes in patients living with Long COVID.

Patients expressed concerns about the challenges in diagnosis and treatment (see Figure 1). A common problem was misdiagnosis, which led to mistrust, delays in treatment and worsening of illnesses: “When the doctor who wanted to refer me to the psychiatrist said, ‘your lungs are perfectly fine,’ I went to the emergency room,[…] and they told me, ‘You have a damaged lung’… So, there you lose trust in healthcare workers” (E16). This was often compounded by the stigmatisation perceived by many participants: “I need to go to the psychiatrist for pain management, but it has been very stigmatizing[…]doctors manage uncertainty very poorly” (E13).

The lack of diagnostic tests left some patients in a legal limbo (unable to get legal rights or benefits, such as insurance or disability compensation, because they do not have an official diagnosis): “When all this broke out, there weren't enough tests, and[…] you don't have a PCR, I can't put it in the report or anything at a legal level” (E18). In some cases, healthcare professionals denied the disease: “The doctor told me, ‘I don't know how they let you in.’ This is for people who are sick (E20). Some participants reported that their symptoms were initially misattributed to other conditions, delaying diagnosis and appropriate treatment, such as menopause. However, the diagnosis was a relief for several participants, validating the patients' experiences: “I felt relieved when I received the diagnosis because until that moment, I was alone. I felt isolated and as if I were losing my mind” (E7).

A lack of patient‐centred care and empathy were the recurrent factors in the healthcare experience. Many participants felt misunderstood by health professionals, describing encounters marked by apathy and a lack of compassion. Nevertheless, positive experiences highlighted the importance of empathy and personalised care: “With that neurologist, perfect, really, I have fallen into wonderful hands, great humanity, great empathy” (E12).

Treatment approaches varied, with some patients receiving pharmacological treatments such as analgesics, cardiovascular drugs and antidepressants. Most felt that these treatments were temporary solutions rather than curative: “Yes, the internist, apart from giving me palliative treatment for the pain, because[…] there's no fix for that” (E18). Concerns about side effects were also common: “They took me off paracetamol because the tests they discovered I had liver damage” (E12). A few participants sought natural remedies: “So, they sent me to the cardiologist[…] but I don't like messing with the heart I already told them. But well, I got something natural, and I'm going to try the natural first, and then if I see that the natural doesn't work, then I'll take those pills they told me” (E10).

Rehabilitation played a crucial role, with cognitive, psychological and speech therapy being effective: “I was in treatment with a speech therapist because I also have dysphagia, and I would say it has improved somewhat” (E19). Physiotherapy and occupational therapy were also beneficial: “The only thing I can say that has helped me a lot is respiratory physiotherapy for the dyspnea. That did help me quite a bit” (E2). However, challenges like post‐exertional fatigue limit their engagement in rehabilitation: “I was doing more respiratory rehabilitation with a private physio, but the effort triggered more fatigue, and it went off the charts” (E13). Access to treatments such as physiotherapy or psychological support was limited for many participants. Most mentioned that these services required long waiting times and were very scarce, so they had to resort to private consultations, bearing the cost personally, making it difficult to access essential therapies.

The Spanish healthcare system is a hybrid, primarily public system that is funded by taxes and offers universal coverage. The National Health System (SNS), which oversees it, offers all resident hospitals, specialised and primary care. Long waiting times in the public system often forced patients to seek faster care despite the high cost: “I have been waiting for nine months for them to see me and do the tests, and that's not the best thing that could happen to you” (E1). “I have been lucky to have private healthcare. It costs me an arm and a leg, but I prefer it because at least I get fast care” (E3). Others preferred the public system for its accessibility and were concerned that privatisation could worsen services: “I'm pro‐public healthcare[…] if you want to eliminate a service, make it precarious first” (E6).

Patients faced not only disparities between public and private healthcare but also a lack of coordination within each system. Many reported that specialists focused solely on specific symptoms without communicating with other professionals, leading to fragmented care and incomplete follow‐ups: “The public hospital was a disaster because I went with private diagnoses, and they didn't believe any of it” (E17). As a result, patients struggled with inconsistent treatment, increased stress and interruptions in their healthcare journey. This fragmentation affected treatment, leading to inconsistent treatment, additional stress and discontinuity in healthcare.

Participants emphasised the need for multidisciplinary care specialised in LC because of the broad combination of symptoms affecting multiple organ systems, which is often unavailable. Some patients sought support from alternative sources, such as patient associations or unrelated groups: “I asked my GP if there were any groups at the outpatient clinic, but since there were none for LC, he told me there were for fibromyalgia and I was going last year” (E19).

#### 3.2.2 Adaptation and Assistance

Patients with LC have encountered adaptation and support needs to cope with the physical and emotional challenges of their symptoms and to reintegrate into daily life and work. Social support networks are crucial, as many experienced isolation due to a lack of understanding of their surroundings. Patient associations have provided a vital source of support and empathy: “Loneliness goes from a sick person alone to a sick person accompanied by others who have the same thing” (E7). Several participants identified the need for adaptations to manage daily activities, particularly mobility, which in many cases restricts independence in both public and private spaces: “I live in a privileged environment… but it has become my green prison. Because when I leave the house, there is a slope… over a hundred stairs” (E12).

Many participants required workplace and schedule adaptations: “In occupational risk prevention, they have to take it seriously and make real adaptations” (E4). The uncertainty and lack of support caused anxiety and distress in some patients: “I had an anxiety attack, because I cannot work[…] I have a job with a lot of responsibility and people under my charge” (E14).

At home, some required assistance from family members: “At first, my husband had to wash my hair sometimes, even my kids” (E19). Cognitive issues also led them to use coping mechanisms: “I've done a university master's degree, but now… many times I have to put notes on myself because I forget things” (E9).

Implementing structured routines and rest periods is essential to prevent exhaustion and manage their energy, though many found a lack of clear medical guidance: “I noticed that I got worse the moment I stopped following my routines” (E9).

#### 3.2.3 Feelings and Emotional Impact

Patients with LC often face feelings of disconnection and misunderstanding. This emotional discomfort is intensified by the stigma and lack of understanding from society and healthcare professionals: “I wish people would show more understanding and empathy. And treat us with more respect” (E4). Social stigmatisation was highlighted: “I don't want a ‘little pension,’ I want to live a full life, as they do. Without having to make sacrifices[…] They should know that there's always a chance of falling into a chronic illness” (E15). Social isolation and the loss of an active social life contribute to their loneliness. Work and economic problems add another layer of stress: “Right now[…] I cannot stand an hour or two working, even if I'm at home with the computer, I cannot do it” (E18).

However, positive emotions like hope, motivation and gratitude surface as a resilience strategy: “I keep hoping for a new treatment,[…] and have a little more QoL. So, that's what keeps me from falling all the way down” (E11). Support from family and friends was also important: “Without my family, I would not have come this far. I am very grateful to them for always being with me” (E6).

### 3.3 Health Information Barriers

#### 3.3.1 Difficulties and Risks of Health Information Seeking

Many patients faced difficulties in accessing reliable information and identifying a mix of sources of information, including social media, medical information and patient associations. The quality of information received from doctors and the updating of doctors' training was another area of concern: “The other day, a doctor, when I went for a test, asked me if I was still COVID‐19 positive.[…] A doctor should be up to date and knowing about new techniques and diseases” (E19). Consequently, most participants preferred seeking information from the internet rather than doctors. “It's not that the doctor doesn't help you. On top of that, it takes away from you and makes you even more confused than you already are” (E17).

Misinformation was an issue mentioned by some participants, who often turned to social media or research articles due to a lack of medical information. Patient associations filtered trustworthy content: “At first, I searched for information on Google because I had very strange sensations, with things I had never felt before. Moreover, I learned a lot through the association of LC. We passed on scientific articles” (E19). However, some participants highlighted the risks of misinformation: “Firstly, when I started connecting with other patients, I did it through Telegram, and there were a lot of false reports, fake news that only made you more anxious” (E18).

Another challenge was information overload, leading some to disconnect from constant updates to protect their mental well‐being: “I've had to move away from reading about it daily. Because information can also be a double‐edged sword. I was overwhelmed by being very informed and seeing nothing that worked for me. I was very aware of reading everything that came out” (E19). Despite the risk, some patient communities played an essential role in filtering trusted content and provided valuable support: “We have a region‐wide chat group with people who have LC. There, we share articles, everything new that comes out, and that's how we stay informed” (E8).

#### 3.3.2 Healthcare Information

Accessing accurate information about LC was complicated by stigma and misunderstandings due to a lack of awareness: “It is a total stigma, if you have cancer or you are missing a leg, it is visible; but when there are no external signs, they don't understand anything” (E17).

Many participants highlighted the absence of detailed, continuous and accessible guidance from healthcare personnel, leading to disorientation and fragmented care. The lack of follow‐up for persistent symptoms created feelings of abandonment and uncertainty. Participants often resorted to online forums, social networks and patients' associations to compensate for insufficient adequate resources from healthcare professionals: “Only those who live with it or those who live next to someone who has it been informed… In other words, people have no idea” (E17).

### 3.4 Quality of Life

LC significantly affected the participants' QoL, causing major disruptions to their daily lives: “Yes, I go with a wheelchair, I have a QoL that is not what I should have with my age… I make the plans I can, enjoy it as much as I can” (E17). Another participant expressed: “To have QoL, you must have a life first. Having a continuous headache… I am surviving” (E14).

**Figure 2:**
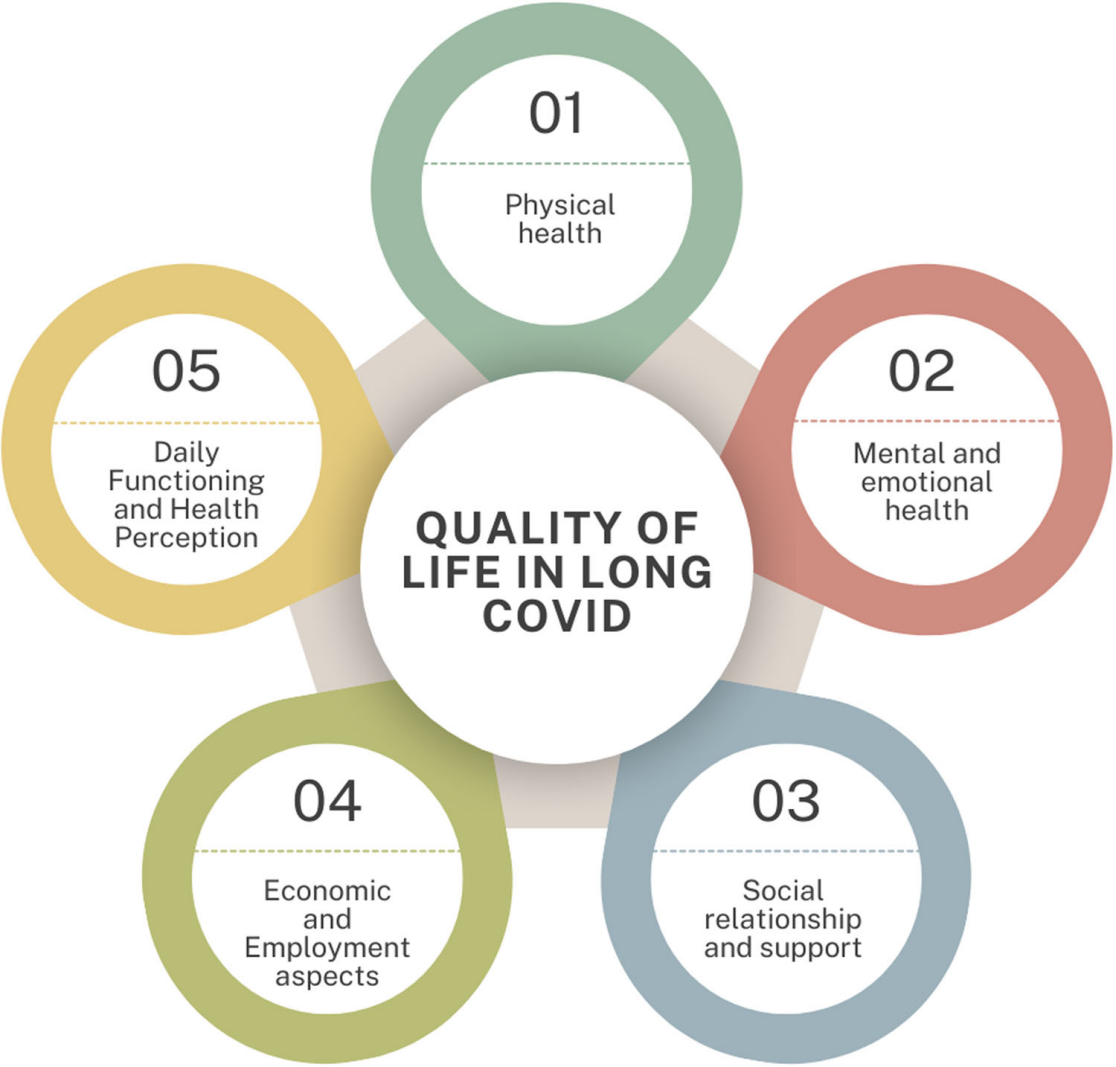
Dimensions of quality of life in LC patients.

Five key dimensions of QoL were identified in LC patients: physical health, mental and emotional health, social relationships and support, economic and employment problems, and daily functioning and health perception (see Figure 2).

#### 3.4.1 Physical Health

Physical health, including pain, physical fatigue and functional abilities, was the most mentioned dimension. Many reported limitations in daily life: “Everything that involves cardiovascular exercise is my problem… There are times when I cannot walk, and my husband must carry me” (E7).

Pain was widespread, particularly migraines, headaches and musculoskeletal pain often rated above 7 on the visual analogue scale. In fact, most of the participants experienced it daily: “Usually, the worst are the headaches… But I also have pain in joints, arms, legs” (E2). Fatigue, especially post‐exertion fatigue, was another prominent issue: “I wake up every day as if I had run 20 kilometers” (E6).

Functional abilities were also compromised, particularly strength and mobility: “I have lost strength, especially in my hands… it's like hanging clothes, my arms get very tired” (E9). Limitations in everyday activities, such as driving or even walking: “It bothered me a lot to drive… because the pain in my arm and all over my side[…] driving is dangerous, and you can't be like that” (E5).

#### 3.4.2 Mental and Emotional Health

LC has also had a significant psychological impact, with patients reporting emotional distress (i.e., anxiety, depression and post‐traumatic stress disorder), mental fatigue and cognitive impairment (such as brain fog and memory problems): “It generates stress, it generates anxiety, worry, a lot of uncertainty because I don't know when I'm going to have an outbreak, I don't know when I'm going to get over it” (E7).

Adjustment disorders and post‐traumatic stress were also reported: “It affects me too, adjustment disorder, as they call it, plus my illness… I am in therapy for a suicide attempt. Not because I couldn't handle my situation, but because… I am taking away the lives of my family” (E12). Mental fatigue was a key issue, with participants reporting cognitive exhaustion triggered by minor stressors. Cognitive and physical fatigue often overlapped, intensifying overall exhaustion: “Not only do I get physically tired, but I also get tired of talking or watching TV” (E18). Cognitive dysfunctions, such as brain fog and memory problems, also affected QoL: “I forgot my son's name… I've had situations where I didn't know if a traffic light was red or green” (E6).

#### 3.4.3 Social Relationships and Support

Social relationships were essential, but LC symptoms and limitations impacted interactions: “I've been to family events and laid down, thinking I need to rest, but it's unpleasant because you don't want to worry people” (E6). Participants emphasised the importance of social and family support, especially from close relatives: “When I cannot walk, my husband has to carry me or move me around the house in an office chair” (E7). Some participants even reported finding new ways to enjoy social connections: “I had never played a board game with my grandparents for so long… You learn to enjoy the time” (E17). However, a few participants mentioned the relationship strain: “Things have changed with my partner, even though we don't argue because I don't have the strength to argue. It's like a fairy tale, but things are much cooler” (E19).

Social isolation was a common experience for many participants: “You find yourself with the four people you can count on. Because illness is scary[…] So, there's no more social life” (E3). Social life also contributed to emotional strain: “People say, ‘How young she is, and she is in a wheelchair,’ and it affects me, I start to cry… it exhausts me both emotionally and physically” (E18).

#### 3.4.4 Economic and Employment Aspects

Financial difficulties and work limitations significantly affected their QoL, with 56.52% of the participants either unemployed or suffering from sick leave. In fact, in most cases, participants attributed their employment status directly to LC, which limited their ability to maintain previous roles or return to work. Four participants had a legally recognised permanent disability. A few lacked financial aid due to legal uncertainty: “Financially, I need them to give me help for everything because I can't even pay for medication[…] I am not receiving any help from the state” (E18).

The limitations and incapacity encountered when performing their work activity were also highlighted: “Right now, I don't feel qualified to be with twenty‐something children…. I often write and say I don't remember this spelling rule, I mean, I'm not here to teach, I'm here to relearn[…] so I wouldn't put up with it at all…” (E18). Some participants improved their health after taking sick leave: “I think I did well on that sick leave because… Well, I started the swimming pool thing[…] Immediately, the pain was reduced” (E20). In addition, several participants noted the high cost of certain treatments: “I go every week to the physio, because otherwise you cannot walk. That's all fine, but because I'm paying for it.”

#### 3.4.5 Daily Functioning and Health Perception

Participants struggled with basic daily activities and independence: “The problem is that when I work, I go from work to bed. All afternoon I have no energy left” (E6). Some participants adapted their routines to maintain autonomy: “To clean[…] I decided not to call anyone to make the effort myself” (E1).

However, even the most basic activities can be challenging for many: “It limits you for the most basic things, for example, I go shopping and I take a bag with my right hand, so that it doesn't weigh too much, and you can't imagine how it hurts… and driving, I live in the outskirts, and I have two daughters to pick up, to take to school… and driving hurts” (E5). Some even need help with basic personal care: “There are days when I can shower by myself, I can lather my hair by myself, but they already have to help me dry myself and I need to lie down for a while before I do my hair” (E12).

Some mentioned that their health perceptions of health deteriorated over time: “I feel sick, because over time you don't recover[…] Look, having fibromyalgia, having migraines, and having… I've never considered myself ill, but now I do consider myself ill. Because I cannot breathe” (E8). In extreme cases, a few participants even contemplated euthanasia due to their condition: “I've become useless, but I've become useless in my body and my head. So, this gives a feeling of not disability, but incapacity and dependence.[…] I determined about euthanasia, and it seems that it is very complicated” (E21).

## 4 Discussion

Findings show that patients with LC face significant barriers in their QoL, access to information and medical care, leading to frustration, mistrust and emotional distress. Fatigue, pain and financial difficulties were the main limitations of daily functioning in Spain. This study also points out gaps in healthcare access, support systems and the availability of rehabilitation resources, while exploring how patients obtain and interpret health information. It offers new ideas into how misinformation, information overload and lack of expert consultation influence patients' decision‐making and well‐being, adding further burdens to their illness and directly affecting their QoL.

Regarding the characteristics of the sample, it was composed of women (91.3%), a higher proportion than the estimated overall prevalence of LC in other studies (60%–80%) [45, 46]. However, this phenomenon has also been reported in previous qualitative research [12, 47]. This could be explained by a greater predisposition of women to participate in qualitative studies [48] and the influence of gender norms on help‐seeking behaviour and health service use [49]. On the other hand, the average age of our sample is comparable to that of previous studies, with a diversity that allows us to capture experiences from different age groups and geographic regions.

In terms of participants' experiences, lack of medical knowledge about LC has led to delays in diagnosis and stigmatisation of patients, which is consistent with previous research [12, 50]. In addition, long waiting lists and fragmented care limit specialised care, which hinders adequate treatment [51]. Previous research has underscored the significance of rehabilitation in LC [52, 53]. Barriers in access to rehabilitation—mainly due to the limited availability of physiotherapy and psychological support in the public system—drive many patients to the private care, worsening their economic situation, despite clinical guidelines recommending a multidisciplinary approach [54, 55, 56]. In this context, several patients reported that their testimonies were not heard. This illustrates a situation of epistemic injustice [57], whereby individuals are discredited due to structural prejudices or the lack of biomedical evidence.

Misattribution of symptoms to other conditions, such as mental health issues, has been documented in the literature [15, 51] and, in this study, was identified as a key barrier that generates mistrust and hinders access to adequate treatment. Despite the official recognition of LC, many patients continue to face the delegitimisation of their symptoms in healthcare and social settings, similar to that observed in diseases such as fibromyalgia or chronic fatigue syndrome [58]. This situation can be understood through the concept of invisible illness, where the lack of objective markers makes symptoms appear unreliable. As a result, the invisibility of physical damage increases discrimination, weakens patients' position in the healthcare system and hinders clinical and social validation [59].

Physical and mental impairments led many patients to request adjustments at work such as reduced working hours, teleworking or even job changes. Previous research also highlighted the economic impact of LC [60], which has guided some patients to situations of precariousness, uncertainty and emotional distress, or even to forced abandonment of the labour market. Some participants reported noticing an improvement after a medical leave of absence due to a decrease in stress. However, a few participants have experienced a “progressive loss of autonomy,” in which, over time, they have required more support for activities that they were previously able to perform without assistance. This phenomenon, which has not been widely addressed in the LC literature, may involve a gradual functional decline that affects both patient independence and family dynamics. In addition, legal uncertainty and the lack of official recognition of the disease have hindered access to sick leave and financial assistance. This problem is especially serious in people with no previous work history, jobs abroad or who are unemployed at the time of diagnosis.

These problems not only limit financial stability and availability of essential resources and exacerbate social inequalities in health. Factors such as income, employment and access to support amplify disparities in LC outcomes [51, 58]. In our sample, five participants were healthcare workers infected while working, illustrating the occupational risk linked to caregiving roles. As most participants were women, this also reflects gendered vulnerability. These findings align with previous research showing how social determinants of health, such as job security, healthcare access and caregiving responsibilities, shape the experience and recovery trajectory of LC [61, 62, 63].

On access to healthcare, a lack of clear information and distrust of health professionals led many participants to seek information online, a common pattern observed in rare and emerging diseases where data are often scarce and contradictory [64, 65]. However, excessive exposure to unfiltered health content increases the risk of misinformation and complicates decision‐making [66]. Similarly, a review of qualitative studies [58] online communities can not only offer valuable support, but also contribute to information overload. Although patient networks help filter information, some participants faced misinformation and information overload, consistent with the literature on digital health and chronic disease [56, 57]. In line with findings from other diseases, our results show that patients with LC also experience emotional distress when trying to find reliable information due to persistent uncertainty. These information‐seeking challenges represent a relatively new factor influencing QoL, as navigating misinformation and the lack of professional guidance adds psychological distress and affects patients' ability to manage their condition effectively. Therefore, we have highlighted the need for continued training of healthcare personnel, the promotion of reliable online information through official channels and improvements in healthcare‐patient communication, to mitigate the possible effects of information overload and misinformation.

Concerning the impact on QoL, this study found a profound impact of LC on QoL, which highlights aspects that have been studied little in the literature [31, 34]. In agreement with previous studies [31, 34, 67], our results highlight significant limitations in key areas such as physical and mental health, social relationships and employment. In contrast to most previous studies, which have focused on standardised questionnaires such as SF‐36 and PAC19‐QoL [34, 68, 69], this work offers a more nuanced perspective through an in‐depth qualitative analysis of patients' lived experiences. Although research such as that of Tíscar‐González et al. [35] has pointed to the importance of return to work and the importance of improved healthcare support, they have not fully addressed the psychological impact of LC in a comprehensive manner. In contrast, our study reveals how persistent symptoms provoke feelings of deep despair, to the point that some patients even contemplate euthanasia. Patients contemplate euthanasia because of the loss of autonomy and suffering for themselves and their family circle. This finding underscores the urgent need for specific mental health and social interventions and a more patient‐centred approach in the management of LC.

Although the social dimension has been recognised as a challenge in previous studies [34, 35], especially because of the isolation of patients with LC, our research provides new insights into patients' coping strategies to adapt to their new reality. Participants described how they modified their social interactions and daily routines to maintain a sense of normalcy, despite physical and cognitive limitations. Some mentioned specific examples, such as adapting social dynamics to board games.

In this regard, many previous studies have taken a cross‐sectional approach and relied on self‐reported data through surveys [68, 70, 71], which limits its ability to capture the complexity of the LC experience. This study addresses previous limitations by employing qualitative methods, allowing for a deeper exploration of patients' evolving needs and challenges. Through the inclusion of patient narratives, this work provides a valuable context for understanding the lived experience of LC beyond the scores on the QoL scales. Given the importance of these findings, future research should delve deeper into the long‐term trajectory of these patients, especially in relation to symptom progression and its impact on QoL over time.

This study has some limitations that should be considered. First, the sample was mostly made up of women, which may have influenced the generalisability of the findings. This may limit the representation of men's experiences with LC. However, this gender bias is common in qualitative studies and could be related to the greater willingness of women to participate in research of this type [48]. Second, although the geographical diversity of the participants allows capturing different realities within Spain, the results are not necessarily extrapolatable to other countries with different health and sociocultural systems. Finally, although various strategies have been used to ensure the reliability and validity of the analysis (such as independent coding by two researchers and iterative discussion), the interpretations may be subject to biases derived from the theoretical framework and the experience of the researchers.

Despite these limitations, this study has several strengths. The qualitative methodology of this study sets it apart and offers a comprehensive insight into the experiences of patients in Spain who have LC. The sample's age and geographic diversity enabled a comprehensive and contextualised understanding of the Spanish situation, pointing out important obstacles such as a lack of medical recognition, false information and trouble getting specialised care. Likewise, by addressing the impact of LC on the dimensions of QoL beyond physical health, such as social relationships and the work environment, this study reveals the psychological impact of LC, including hopelessness and contemplation of euthanasia. Patients' coping skills indicate changes they make to their daily routines and social interactions to preserve a certain level of normality. Future research should focus more on how symptoms change over time and on the effectiveness of different rehabilitation techniques. It would also be crucial to evaluate how economic and social support programmes affect patients' QoL. Finally, investigating how both medical professionals and LC patients themselves view and manage information would provide valuable insights for improving care and decision‐making.

## 5 Conclusion

In conclusion, this research shows that LC has a detrimental effect on patients' QoL and leads to financial hardship due to job instability and medical expenses. Medical scepticism and the misattribution of symptoms to psychological reasons intensify emotional discomfort and delay diagnosis and treatment. Social relationships also deteriorate, largely due to isolation linked to the stigma surrounding LC and the physical limitations that restrict participation in daily activities. Patients visit multiple specialists without comprehensive follow‐up due to the fragmentation of the healthcare system. LC patients who rely too heavily on online resources for health information, often due to a lack of expert advice, are exposed to false information and confusion.

Our findings emphasise the need for integrated, multidisciplinary care models that ensure fair access to treatment and improved health communication. Further research is required to refine intervention strategies and evaluate the impact of information that is understudied.

## Author Contributions

**Esther Ortega‐Martin:** conceptualization, methodology, formal analysis, investigation, writing – original draft, writing – review and editing, visualization. **Javier Alvarez‐Galvez:** conceptualization, methodology, investigation, writing – review and editing, supervision, funding acquisition.

## Disclosure

Permission to reproduce material from other sources: Not applicable.

## Ethics Statement

The studies involving humans were approved by the Ethics Committee of Cadiz (PEIBA 0659‐N‐23). The studies were conducted in accordance with the local legislation and institutional requirements.

## Consent

The participants provided their written informed consent to participate in this study.

## Conflicts of Interest

The authors declare no conflicts of interest.

## Supporting information

Supporting Material 1.

Supporting Material 2.

## Data Availability

The data sets analysed in this study contain sensitive patient information and are subject to privacy regulations. They are not publicly available but can be requested from the corresponding author. Access requires prior ethical approval and compliance with confidentiality guidelines.
