# Supplementary material for: Living With Long COVID: Everyday Experiences, Health Information Barriers and Patients' Quality of Life
Source: Health Expect. 2025 May 14;28(3):e70290. doi: 10.1111/hex.70290 (PMC12076351; doi:10.1111/hex.70290)
Supplement: Supplementary file 1 — Supporting Material 1. [file HEX-28-e70290-s001.docx]

Supplementary Material 1

# Guide to Semi-structured Interview

**Theme 1. Quality of life**

- How was your health before Long COVID?
- Considering your current quality of life, what specific aspects or areas would you highlight as most important in your daily life?
- Do you feel pain or fatigue in your daily life?
  - Physical health status
  - How do you feel about it?
- Has your vitality decreased?
- What symptoms do you have? How have they evolved?
  - Previous illnesses
  - Are the symptoms the same since the onset?
- How has Long COVID affected your work or daily life?
  - Previous illnesses, how did you feel.
  - Limitations in your daily life: pain, fatigue, changes in the environment
  - Social activities
- How would you describe your overall health?
- How has Long COVID affected your health?
  - Mental and emotional health
  - Physical health
  - Social activities
- Has your life been socially affected?
  - How do you feel about it?
  - Support received.

**Theme 2. Personal experience and perceived needs**

- What has been the most difficult thing to deal with regarding Long COVID?
- What needs would you say you have at this moment related to your health and well-being?
- Have you received any support that has been helpful during your experience?
  - Do you require support for your daily activities?
  - Type of support received and required.
  - Who or what facilitates that support?
  - Do you have a telecare button?
- What would you like people to know about your experience with Long COVID?
- Is there anything else you would like to add about your experience with Long COVID?

**Theme 3. Use of health services**

- Have you needed healthcare since you have had Long COVID? How has your experience been?
  - Type of care required (emergency, health centre, hospital)
  - How do you feel about the care received?
- Have you needed any medical review by a specialist?
- What specialist?
- How was it?
- Have you received any type of treatment? How has your experience been with this/these treatments?
  - Treatment duration
  - Utility

**Theme 4. Information**

- Has the information received about Long COVID been useful to you?
- What do you think of the information received from healthcare personnel? Is it useful?
- Do you think the information conveyed in the media is reliable?
  - Social networks
  - Television
  - Internet
  - Videos
  - Press

**Anything else to add in general?**
