# Supplementary material for: Living With Long COVID: Everyday Experiences, Health Information Barriers and Patients' Quality of Life
Source: Health Expect. 2025 May 14;28(3):e70290. doi: 10.1111/hex.70290 (PMC12076351; doi:10.1111/hex.70290)
Supplement: Supplementary file 2 — Supporting Material 2. [file HEX-28-e70290-s002.docx]

Supplementary Material 2

# Basic Health Questionnaire

Age:

Height:

Weight:

Sex:

Place of residence (City and province):

- Do you have any diagnosed illnesses? Please specify which ones.
- Do you have any recognized disabilities? What percentage?
- What medications do you usually take?
- What is your current employment status? What was your employment status before having Long COVID?
- How many members are in your household?
- How many people do you live with?
- Do you receive any type of assistance from family, caregiver, or do you have a telecare button? Please specify (A telecare button is an emergency device that allows a person to call for immediate help at the press of a button, commonly used in the care of older or dependent individuals).
- Indicate your level of education
  - Basic studies
  - Primary/ Elementary
  - Secondary/ High school
  - Baccalaureate/ Pre-University
  - Vocational training
  - Bachelor's degree
  - Master's degree
  - Doctorate
  - Others (specify)
- What is your average family income? (net income)
- Have you experienced pain in the last 4 weeks?
- Do you experience pain in your daily life? On a scale of 0 for no pain to 10 for the worst imaginable pain, how much would you say it hurts?
- When did you have COVID-19 resulting in Long COVID?
- Considering healthcare visits, not medical tests, how many visits have you made to your general practitioner in the last twelve months?
- How many visits have you made to a specialist in the last twelve months?
- How many visits have you made to the emergency room in the last twelve months?
- Did any of these visits result in hospitalization?
